# Efficacy and Safety of Continuous Paravertebral Block after Minimally Invasive Radical Esophagectomy for Esophageal Cancer

**DOI:** 10.1155/2020/3105874

**Published:** 2020-04-21

**Authors:** Shifa Zhang, Hongfeng Liu, Haibo Cai

**Affiliations:** Department of Thoracic, Affiliated Jining No. 1 People's Hospital of Jining Medical University, Jining Medical University, Jining, Shandong, China

## Abstract

**Objective:**

To compare the effects of continuous paravertebral block analgesia and patient-controlled intravenous analgesia after minimally invasive radical esophagectomy for esophageal cancer and their effects on postoperative recovery.

**Methods:**

A retrospective analysis was performed among 233 patients who underwent minimally invasive esophageal cancer radical operation and met the requirements, including 87 patients (group C) who were successfully placed with a continuous paravertebral block device under direct vision and 146 patients (group P) who used a patient-controlled intravenous analgesia device. Visual analogue pain score (VAS) at rest and in motion for 1, 3, 6, 12, 24, 36, and 48 hours after awakening, incidence of adverse reactions of the two analgesic methods, occurrence of pulmonary complications after operation, use of emergency analgesics, and hospital stay after operation was recorded.

**Results:**

The VAS scores of group C in resting and active state at 1, 3, 6, 12, 24, 36, and 48 hours after operation were significantly lower than those of group P (*P* < 0.001). The incidence of adverse reactions, pulmonary complications, and the use of emergency analgesics in group C were lower than those in group P (*P* < 0.05). The hospitalization time of group C was significantly shortened, and the satisfaction degree of group C was significantly higher than that of group P (*P* < 0.05).

**Conclusion:**

Paravertebral block is safe and effective for patients undergoing minimally invasive radical esophagectomy. The incidence of adverse reactions and complications is lower, and the satisfaction of postoperative analgesia is higher, which is beneficial to the rapid recovery of patients after operation.

## 1. Introduction

China is one of the countries with the highest incidence and mortality of esophageal cancer in the world [1]. Surgery is the preferred treatment for the early and middle-stage esophageal cancer [2].

Traditional open resection of esophageal cancer is considered as one of the most painful surgical procedures. In recent years, with the development of minimally invasive surgical technology, the trauma of esophageal cancer patients has been greatly reduced [3]. However, severe pain still exists after operation, which is mainly related to the stimulation of the thoracic drainage tube and intercostal nerve traction or injury. The severe and lasting pain after operation not only limits the patient's cough and expectoration, leads to respiratory secretion retention, and causes complications such as hypoxemia, atelectasis, and pulmonary infection, but also induces severe stress response and inhibits immune function [4]. Therefore, good postoperative analgesia has positive significance for patients' rapid recovery and perioperative safety [5]. In recent years, thoracic paravertebral nerve block technology has been more and more used for postoperative analgesia of esophageal cancer [6]. A subcutaneous analgesic device modified by our center is a continuous paravertebral analgesia (CPVB) device, which is placed under intraoperative thoracoscopy. In this study, we compared the analgesic effect of the device with that of patient-controlled intravenous analgesia (PCIA) and explored the efficacy and safety of the improved analgesic device in clinical application. In group C, instead of opioid analgesics, which are used in group P, some drugs are used for postoperation analgesia, such as ropivacaine, bupivacaine, and lidocaine hydrochloride.

## 2. Methods

### 2.1. General Information

A retrospective analysis was performed among the patients who underwent radical esophagectomy in our hospital from December 2016 to December 2017. Inclusion criteria were (1) age ranged from 20 to 80. (2) The American Society of Anesthesiologists (ASA) is graded I or II. (3) Minimally invasive radical esophagectomy was performed in our hospital. (4) Clear consciousness, after explaining, can independently describe and evaluate pain. (5) The relevant research data are complete. Exclusion criteria: (1) abnormal coagulation function. (2) Analgesics, anesthetic allergies, or addictions. (3) Taken sedative and analgesic drugs in recent 3 months. (4) There is a history of chronic pain. (5) Morbid obesity (body mass index (BMI) > 35 kg/m^2^) or thoracic deformity. (6) Infection at the puncture site. The patients were divided into two groups according to different analgesic schemes: continuous paravertebral nerve block analgesia group (group C) and PCIA group (group P). This study was informed by the Hospital Ethics Committee. A total of 233 patients were enrolled in this study, including 87 in group C and 146 in group P. There was no significant difference in baseline data of sex, age, height, weight, and operation time between the two groups (*P* > 0.05), as shown in Table 1. In group C, puncture bleeding occurred in 1 case (1.1%) during the operation of thoracic paravertebral nerve block under direct vision. After hemostasis, catheterization was successfully placed in the paravertebral space of T5. Puncture of pleura occurred in 3 cases (3.4%). Catheterization was successful after replacing the puncture site. No pneumothorax, local anesthetic poisoning, or other puncture complications occurred. The analgesic pump and catheter were removed in group C after 72 hours of analgesic treatment. The analgesic pump was removed 48 hours after operation in group P.

### 2.2. Paravertebral Thoracic Block

Patients were admitted to the hospital for routine examination, and the operation was performed within a limited time after the contraindication was eliminated. Pulmonary function exercise and health education were done before operation. All patients received rapid induction of intravenous inhalation combined with general anesthesia through oral single-lumen endotracheal intubation. After thoracic surgery, the same group of surgeons performed paravertebral nerve block puncture and catheterization under thoracoscopy. The steps are as follows: (1) selection of puncture site and paravertebral space of T6. (2) Puncture needles were inserted into the patient's back to the parathoracic space under the direct vision of thoracoscopy or the Da Vinci robotic surveillance system. (3) Given 0.375% ropivacaine 20 mL of loading dose, the pleural wall was protruded and was intact. (4) The catheter was sent to the space under thoracoscopic direct vision. (5) The catheter was fixed properly. A continuous analgesia pump was connected after operation. Group P was connected with a patient-controlled intravenous analgesia pump after operation. After the vital signs were stable, the tracheal intubation was removed and 25% oxygen was inhaled. Postoperative management is carried out by a unified trained and experienced senior physician.

### 2.3. Preparation of Analgesic Pump

Patients in group C were given an improved paravertebral nerve block analgesia scheme. Drugs in the analgesic pump were formulated as follows: ropivacaine 300 mg + bupivacaine 75 mg + lidocaine hydrochloride 400 mg. The above medicines were prepared into 150 mL solution with normal saline at a pump speed of 2 mL/h. Local anesthesia concentration was 0.2% ropivacaine, 0.05% bupivacaine, and 0.26% lidocaine hydrochloride, respectively. Patients in group P were given PCIA. Drug formulation in the analgesic pump: dexmedetomidine 200 mg + sufentanil 50 *μ*g + digoxin 20 mg + alosetron 10 mg. The intravenous infusion was carried out at a sustained dose of 2 mL/h.

### 2.4. Observation Indicators

The observation indicators are as follows: (1) visual analogue scale (VAS) after operation and pain score in resting and active state at 1, 3, 6, 12, 24, 36, and 48 hours after conscious extubation of tracheal intubation in both groups; (2) adverse reactions related to analgesia (nausea, vomiting, dizziness, sleepiness, and itching of skin); (3) pulmonary complications after operation; (4) number of emergency analgesic drugs used within 48 hours after operation; (5) patient satisfaction with analgesia (according to very satisfactory, general, and unsatisfactory evaluation, very satisfactory and satisfactory were calculated into the satisfaction rate); (6) prognostic indicators (the first time out of bed after operation, the time of thoracic tube removal after operation, and the time of hospitalization after operation, etc.).

### 2.5. Statistical Methods

SPSS22.0 statistical software was used to analyze the data. The measurement data were expressed as mean ± standard deviation, and the independent sample *t*-test was used for comparison between groups. The chi-square test or the Fisher exact probability method were used to compare the counting data. The difference was statistically significant with *P* < 0.05.

## 3. Results

### 3.1. Comparison of VAS Scores at Different Time Points after Operation

The VAS scores of resting and active state pain in group C at 1, 3, 6, 12, 24, 36, and 48 hours after conscious extubation of tracheal intubation were lower than those in group P (*P* < 0.001), and the difference was significant (*P* < 0.001), as shown in Tables 2 and 3.

### 3.2. Adverse Reactions and Overall Satisfaction

In group C, 2 patients (2.3%) had pain and discomfort at the parathoracic cannula after operation, and the pain relieved spontaneously without nausea, vomiting, and dizziness. In group P, 18 patients (12.3%) had nausea, vomiting, and dizziness after operation, and the symptoms improved after stopping the analgesic pump. Two patients (2.3%) in group C were complicated with atelectasis or pulmonary infection, while 14 patients (9.6%) in group P were complicated with atelectasis or pulmonary infection. In group P, 23 patients (15.8%) were given analgesics after operation, while only 3 patients (5.7%) in group C. The overall satisfaction of analgesia was 95.4% in group C and 76.7% in group P. There was a significant difference between the two groups in the above items (*P* < 0.05).

### 3.3. Postoperative Hospitalization Indicators

The first time of getting out of bed and the time of removing the thoracic duct in group C were earlier than those in group P, and the hospitalization time was significantly shorter and the hospitalization cost was lower, as shown in Table 4.

## 4. Discussion

Pain is a ubiquitous and unavoidable problem in surgery, and the pain caused by thoracic surgery is particularly common and severe [7]. Most patients have persistent pain after surgery, and the incidence of chronic pain after surgery is very high. The severity of acute pain after surgery is one of the causes of chronic pain after surgery [8]. However, with the development and popularization of minimally invasive techniques in thoracic surgery, the pain of patients after surgery is less than that of traditional thoracotomy [9]. After esophagectomy because of the compression, traction after the placement of drainage tube, and the patient's breathing movement, the wound will be excessively painful, so that patients reject spontaneous breathing, protectively reduce breathing movements, and reduce tidal volume, and at the same time, the respiratory frequency increases compensatively and oxygen diffusion ability decreases, resulting in postoperative hypoxemia [10]. Studies have shown the incidence of acute respiratory failure after esophageal cancer surgery, which is one of the main causes of death after esophageal cancer surgery [11]. In addition, intraoperative lung traction, pleural effusion, and other factors can also cause pulmonary atelectasis, which increases the incidence of pulmonary infection [12]. Therefore, it is necessary to deal with the pain after operation actively and effectively.

At present, there are morphine analgesia, epidural analgesia, PCIA, thoracic paravertebral block analgesia (TPVB), and so on [13]. Morphine can inhibit the respiratory and immune functions of the body and lead to the growth and metastasis of residual tumor cells [14]. Epidural analgesia can effectively relieve postoperative pain without respiratory inhibition, but epidural block analgesia has potential complications such as epidural perforation, epidural hematoma, and epidural infection [15]. High-epidural block can inhibit myocardium, dilate blood vessels in the blocked area, and is prone to circulatory inhibition [16]. PCIA is widely used in clinics due to easy application and nurse. However, high concentration of anesthetics can inhibit breathing and cause nausea, vomiting, and other adverse reactions; and the effect of early analgesia after operation is not ideal [17]. TPVB is a nerve block technique that injects local anesthetics into the thoracic spinal nerve from the perforation of the intervertebral foramen, i.e., the paravertebral space of the thoracic spine, and thus produces anesthetic effects on the somatic and sympathetic nerves at the same side of the injection site [18]. TPVB can successfully anesthetize the front, side, and back of the thorax and abdomen and effectively block the sensory, motor, and sympathetic nerve fibers of the thoracic segment. Traditional parathoracic block by blind exploration through the classical approach can easily damage the pleura and cause damage to blood vessels and nerves. At present, ultrasound-assisted paravertebral nerve block is often used clinically, which improves the accuracy of localization and reduces the complications such as epidural diffusion, pneumothorax, and nerve injury [19]. However, it is still less safe and is simple, direct, and accurate in operation than that under direct vision.

With the popularization and development of video-assisted thoracoscopy, clear vision and operating environment provide convenience for paravertebral nerve block under direct vision. We modified a continuous paravertebral analgesia device suitable for patients undergoing thoracic surgery and placed under direct vision during the operation [20]. The device has a set of simple and easy operation process. It has the following characteristics: (1) the operation monitoring system can obtain intrathoracic vision and guide the puncture needle into the paravertebral space of T6 under direct vision, which is safe and reliable. (2) The success of puncture or catheterization can be judged by injecting 0.2% ropivacaine hydrochloride or saline directly and observing whether the pleural wall is protruded at the designated position. (3) The length of the improved catheter is suitable, and the design of the osmotic catheter with a flexible front end and side hole can avoid puncturing the pleura and catheter blockage. (4) The connected paravertebral continuous analgesia pump is light and easy to carry, and it is convenient for patients to move early after operation. Studies have shown that the sensory block level of single T6 level paravertebral block can reach 9 to 10 segments after 20 mL mepivacaine injection. In this study, only 1 case of puncture bleeding and 3 cases of punctured pleura were present.

After hemostasis, replacement of puncture position, and successful catheterization, there was no pneumothorax, local anesthetic poisoning, and other complications. All patients using the CPVB program can confirm the correct position of the catheter under direct vision, with strong reliability. The improved paravertebral analgesic catheter has the characteristics of anterior disc curvature, lateral foramen distribution, and hemispherical tip design with fine foramen, which can better perform the continuous paravertebral nerve block. In addition, the formulation of local anesthesia drugs in the analgesic pump refers to the relevant literature. The formulation of 0.2% ropivacaine, 0.05% bupivacaine, and 0.26% lidocaine hydrochloride has a good effect of continuous analgesia. The total volume of the analgesic pump is 150 mL, and the continuous pumping speed is 2 mL/h, which can meet the needs of 72 hours after operation. The VAS score of resting and active state pain in group C was lower than that in group P, and the incidence of adverse reactions such as nausea, vomiting, and dizziness were lower, the use of emergency analgesics including opioids was less, the satisfaction of general analgesia was higher, the incidence of pulmonary complications such as atelectasis and pneumonia after operation was lower than that in group P, and the early out-of-bed activity and various hospitalization indicators were better than those in group P. The good effect of continuous analgesia after operation in group C alleviated the pain of patients in the resting and active state, enabled patients to get out of bed early, and cough and sputum effectively, which was conducive to the early recovery of respiratory function, thus reducing the occurrence of pulmonary complications, which was also conducive to the recovery of the body and immune function of patients after operation, promoting the rapid recovery of patients after operation, and thus reducing the hospital stay after operation, interim, and cost. In group C, instead of opioid analgesics, some nonopioid drugs are used for postoperation analgesia, including ropivacaine, bupivacaine, and lidocaine. The postoperative emergence use opioid analgesics in group C was also lower than that in group P. In terms of safety, using less frequency and lower dose of opioid analgesics is the best advance for safer opioid analgesia strategies.

This shows that our improved continuous paravertebral nerve block analgesia device is superior to the patient-controlled analgesia by intravenous infusion and has the advantages of longer indwelling time (72 hours) than PCIA, easy to carry, and so on.

In conclusion, the modified continuous paravertebral nerve block device under direct vision in minimally invasive esophageal cancer radical operation is safe, simple, direct, and accurate, and the effect of continuous analgesia after operation is stable, and the rate of adverse reactions is low, which is conducive to the rapid recovery of patients after operation.

## Figures and Tables

**Table 1:** Comparison of baseline data and surgical conditions between two groups.

Characteristics	Group C (*n* = 87)	Group P (*n* = 146)	*P* value
Gender			0.899
Male	54	93	
Female	33	53	
Age	63.94 ± 6.35	65.32 ± 7.84	0.165
Height (cm)	166.10 ± 7.85	167.20 ± 9.01	0.346
Weight (kg)	65.49 ± 10.39	63.99 ± 11.24	0.312
Time of operation (min)	285.20 ± 52.10	294.70 ± 49.20	0.312
Intraoperative blood loss (ml)	327.40 ± 54.07	336.30 ± 48.92	0.198
Tumor size (cm)	3.45 ± 1.38	3.72 ± 1.85	0.248
Tumor location			0.31
Upper segment	18	25	
Middle segment	56	107	
Lower segment	13	14	
TNM staging			0.421
I	59	110	
II	11	16	
III	17	20	

**Table 2:** Comparison of VAS scores of the resting state at different time after operation in two groups.

Time (h)	Group C (*n* = 87)	Group P (*n* = 146)	*P* value
1	1.9 ± 1.2	3.2 ± 1.1	<0.001
3	2.4 ± 1.4	3.4 ± 1.6	<0.001
6	2.2 ± 1.3	3.7 ± 1.5	<0.001
12	2.3 ± 1.1	3.1 ± 1.0	<0.001
24	2.2 ± 1.2	3.0 ± 1.6	<0.001
36	2.0 ± 1.2	2.9 ± 1.4	<0.001
48	1.8 ± 1.4	2.8 ± 1.8	<0.001

**Table 3:** Comparison of VAS scores of the active state at different time after operation in two groups.

Time (h)	Group C (*n* = 87)	Group P (*n* = 146)	*P* value
1	2.2 ± 0.8	3.4 ± 1.2	<0.001
3	3.2 ± 1.8	3.8 ± 1.8	<0.001
6	3.4 ± 1.6	4.2 ± 1.7	<0.001
12	3.1 ± 1.2	4.0 ± 1.4	<0.001
24	3.0 ± 1.1	3.8 ± 1.2	<0.001
36	26 ± 1.1	3.5 ± 0.9	<0.001
48	2.3 ± 1.2	3.4 ± 1.5	<0.001

**Table 4:** Comparison of postoperative hospitalization indicators between the two groups.

Indicators	Group C (*n* = 87)	Group P (*n* = 146)	*P* value
Time of first ambulation after operation (h)	19.4 ± 4.6	36.3 ± 7.2	<0.001
Time of thoracic tube removal (d)	3.7 ± 0.7	5.2 ± 1.4	<0.001
Postoperative hospital stay (d)	8.9 ± 1.8	11.8 ± 2.4	<0.001

## Data Availability

The data underlying the findings of the study could be obtained if a request is sent to the corresponding author at 13518670801@163.com.
